# 

**Published:** 1851-11

**Authors:** 


### WESTERN JOURNAL

OF

### MEDICINE AND SURGERY.

### EDITORS.

---

**LUNSFORD P. YANDELL, M. D.**

PROFESSOR OF PHYSIOLOGY AND PATHOLOGICAL ANATOMY IN THE UNIVERSITY OF LOUISVILLE.

AND

**THEODORE S. BELL, M.D.**

### RECEIPTS OF MEDICAL JOURNAL FOR OCTOBER, 1851.

| | |
|---|---|
| Drs. Pollock & Savage, Bardstown, Ky., | 10 00 |
| Dr. J. M. Wells, Washington, Ind., | 10 00 |
| —— Tully, Mt. Carmel, Ky., | 5 00 |
| J. H. Donell, Franklin, Ind., | 10 00 |
| J. Mitchell, Steubenville, Ohio, | 5 00 |
| J. M. Larkin, Charlotte, Tenn. | 5 00 |
| M. M. Shepherd, Newbern, Ind., | 5 00 |
| C. C. Smith, Richmond, Ky., | 10 00 |
| G. W. Wheeler, Perryville, Mo., | 5 00 |
| J. Slavens. Portland Mills, Ind., | 10 00 |
| W. R. Chew, Midway, Ky., | 5 00 |
| P. J. Snead, Alexandria, Penn., | 5 00 |
| J. M. Baird, Shady Grove, Penn., | 5 00 |
| J. R. Bettison, Louisville, Ky., | 5 00 |
| S. N. Hall, Paris, Ky., | 5 00 |

